# Driving under the influence of alcohol: frequency, reasons, perceived risk and punishment

**DOI:** 10.1186/s13011-015-0007-4

**Published:** 2015-03-12

**Authors:** Francisco Alonso, Juan C Pastor, Luis Montoro, Cristina Esteban

**Affiliations:** DATS (Development and Advising in Traffic Safety) Research Group, INTRAS (University Research Institute on Traffic and Road Safety), University of Valencia, Serpis 29, 46022 Valencia, Spain; FACTHUM.lab (Human Factor and Road Safety), INTRAS (University Research Institute on Traffic and Road Safety), University of Valencia, Serpis 29, 46022 Valencia, Spain

**Keywords:** Drivers, Road safety, Driving while intoxicated, Driving under the influence, Alcohol

## Abstract

**Background:**

The aim of this study was to gain information useful to improve traffic safety, concerning the following aspects for DUI (Driving Under the Influence): frequency, reasons, perceived risk, drivers' knowledge of the related penalties, perceived likelihood of being punished, drivers’ perception of the harshness of punitive measures and drivers’ perception of the probability of behavioral change after punishment for DUI.

**Methods:**

A sample of 1100 Spanish drivers, 678 men and 422 women aged from 14 to 65 years old, took part in a telephone survey using a questionnaire to gather sociodemographic and psychosocial information about drivers, as well as information on enforcement, clustered in five related categories: “Knowledge and perception of traffic norms”; “Opinions on sanctions”; “Opinions on policing”; “Opinions on laws” (in general and on traffic); and “Assessment of the effectiveness of various punitive measures”.

**Results:**

Results showed around 60% of respondents believe that driving under the influence of alcohol is maximum risk behavior. Nevertheless, 90.2% of the sample said they never or almost never drove under the influence of alcohol. In this case, the main reasons were to avoid accidents (28.3%) as opposed to avoiding sanctions (10.4%). On the contrary, the remaining 9.7% acknowledged they had driven after consuming alcohol. It is noted that the main reasons for doing so were “not having another way to return home” (24.5%) and alcohol consumption being associated with meals (17.3%).

Another important finding is that the risk perception of traffic accident as a result of DUI is influenced by variables such as sex and age. With regard to the type of sanctions, 90% think that DUI is punishable by a fine, 96.4% that it may result in temporary or permanent suspension of driving license, and 70% that it can be punished with imprisonment.

**Conclusions:**

Knowing how alcohol consumption impairs safe driving and skills, being aware of the associated risks, knowing the traffic regulations concerning DUI, and penalizing it strongly are not enough. Additional efforts are needed to better manage a problem with such important social and practical consequences.

## Background

In Europe, traffic accidents are one of the main causes of mortality in people between 15 and 29 years old, and driving under the influence of alcohol (DUI) is a major risk factor in most crashes [1,2].

In the year 2001 in Spain, 40,174 people were treated in public hospitals for traffic injuries. Some 28% of these injuries were serious or very serious and drinking was involved in a high percentage of cases. According to the Spanish Directorate General of Traffic (DGT), alcohol is involved in 30-50% of fatal accidents and in 15 to 35% of crashes causing serious injury, constituting a major risk factor in traffic accidents. This problem is especially important among young people and worsens on weekend nights [3,4].

In more recent years, several studies have shown that more than a third of adults and half of teenagers admit they have driven drunk. We also know that most of them were not detected. Generally, the rate of arrests for driving under the influence is very low and even those drivers who were arrested were mostly “first-time” offenders [5].

Some studies show that many young people lack information or knowledge about the legislation regulating consumption of alcohol for drivers, as well as the effects of this drug on the user [6-8].

There are also some widespread beliefs and misconceptions regarding the actions the driver can take in order to neutralize the effects of alcohol before driving (for instance drinking coffee, having a cold shower or breathing fresh air). As suggested by Becker’s model of health beliefs [9,10], preventive behavior is unlikely to occur unless the subject considers the action necessary, hence the importance of providing adequate information and disproving false beliefs.

Drivers are not usually aware of the risk they assume when they drive under the influence of alcohol, as they do not suffer a traffic accident every time they drink and drive. Hence they tend to think there is no danger in driving under the influence of alcohol, incurring the same risk behavior once and again.

But the reality is quite different. Alcohol causes very obvious alterations in behavior, as it affects almost all the physical skills we need for safe driving. It can interfere with attention, perceptual functioning and motor skills, as well as in decision making while driving.

Drinking impairs the ability to drive and increases the risk of causing an accident. The effects of alcohol consumption on driving-related functions are modulated by some factors, such as form of consumption (regular or infrequent), expectations about their consumption, expertise in driving and driver’s age. The increased risk of accident starts at a lower blood alcohol level when drivers are inexperienced or they are occasional drinkers, and begins at a higher blood alcohol level when these are more experienced drivers or regular drinkers [11,12].

The BAC represents the volume of alcohol in the blood and is measured in grams of alcohol per liter of blood (g / l) or its equivalent in exhaled air.

Any amount of alcohol in blood, however small, can impair driving, increasing the risk of accident. Therefore, the trend internationally is to lower the maximum rates allowed.

After drinking, the rate of alcohol in blood that a driver is showing can vary widely due to numerous modulating variables. Among them, some important factors are the speed of drinking, the type of alcohol (fermented drinks such as beer or wine, or distilled beverages like rum or whisky) or the fact of having previously ingested some food, as well as the age, sex or body weight. Ideally, if everyone drank alcohol responsibly and never drove after drinking many deaths would be avoided. Accurate information about how driving under the influence effects traffic safety would be a positive step towards this goal.

### Study framework

Research on enforcement of traffic safety norms has a long tradition. In 1979, a classic work [13] showed that increasing enforcement and toughening sanctions can reduce accidents as an initial effect, although the number of accidents tends to normalize later.

Justice in traffic is needed insofar as many innocent people die on the roads unjustly. This is our starting point and our central principle. In order to prevent traffic accidents, a better understanding is needed of the driver’s knowledge, perceptions and actions concerning traffic regulations. Drivers have to be aware of how important rules are for safety. The present study comes from a broader body of research on traffic enforcement, designed to develop a more efficient sanctions system [5,14].

Our research used a questionnaire to gain sociodemographic and psychosocial information about drivers, as well as additional information on enforcement clustered in five related categories: “Knowledge and perception of traffic norms”; “Opinions on sanctions”; “Opinions on policing”; “Opinions on laws” (general ones and traffic laws in particular); and “Assessment of the effectiveness of various punitive measures”.

A number of additional factors were also explored, including: driving too fast or at an improper speed for the traffic conditions, not keeping a safe distance while driving, screaming or verbal abuse while driving, driving under the influence, smoking while driving, driving without a seat belt and driving without insurance. For a more complete review, see the original study [14].

### Objective

The aim of this study was to gain useful information to improve traffic safety, concerning the following aspects:

- Frequency of driving under the influence of alcohol (DUI).
- Reasons for either driving or not driving under the influence (DUI).
- Perceived risk of DUI.
- Drivers’ knowledge of DUI-related penalties.
- The perceived likelihood of being punished for DUI.
- Drivers’ perception of the harshness of punitive measures for DUI.
- Drivers’ knowledge of the penalties for DUI.
- Drivers’ perception concerning the probability of behavior change after punishment for DUI.
- Sociodemographic and psychosocial factors related with alcohol consumption and driving.

## Methods

### Participants

The sample consisted of 1100 Spanish drivers: 678 men (61.64%) and 422 women (38.36%), between 14 and 65 years of age. The initial sample size was proportional by quota to segments of Spanish population by gender and age. The number of participants represents a margin of error for the general data of ± 3 with a confidence interval of 95% in the worst case of p = q = 50%; with a significance level of 0.05.

Drivers completed a telephone survey. 1100 drivers answered interviews, and the response rate was 98.5%; as it was a survey on social issues, most people consented to collaborate.

### Procedure and design

The survey was conducted by telephone. A telephone sample using random digit dialing was selected. Every phone call was screened to determine the number of drivers (aged 14 or older) in the household. The selection criteria were possession of any type of driving license for vehicles other than motorcycles and driving frequently. Interviewers systematically selected one valid driver per home. The survey was carried out using computer assisted telephone interview (CATI) in order to reduce interview length and minimize recording errors, ensuring the anonymity of the participants at all times and emphasizing the fact that the data would be used only for statistical and research purposes. The importance of answering all the questions truthfully was also stressed.

In this article, we present the data on driving under the influence of alcohol. The first question raised was: *How often do you currently drive after drinking any alcoholic beverage?* Possible responses were: Almost always, Often, Sometimes, Rarely or Never.

If they answered either Almost always, Often or Sometimes, they were asked: *What is the reason that leads you to drive under the influence?* If they answered Rarely or Never, they were asked: *What is the reason you rarely or never drive under the influence?* In both cases, respondents had the option of an open answer.

Later they were asked to rate from 0 to 10 the risk that driving under the influence of alcohol can cause a traffic accident in their opinion (0 being the minimum risk and 10 the maximum risk of crash).

Then they were asked to rate from 0 to 10 the harshness with which they thought DUI sanctions should be administered.

They were also asked: *Is driving exceeding alcohol limits punishable?* In this case, participants had the chance of answering *Yes* or *No*. We would then compare the correct answers with the standard to determine the knowledge.

Drivers who were unaware that DUI is punishable were asked about the probability of being sanctioned for this reason using the following question: *When driving exceeding the limits of alcohol, out of 10 times, how many times is it usually sanctioned?*

Another question dealt with the type of penalties. The participants were asked if the penalties for DUI consisted of economic fines, imprisonment or license suspension, either temporary or permanent. The question raised was: *Have you ever received any penalty for driving under the influence?* Possible answers were *Yes* or *No*. Those drivers who answered affirmatively were then asked about the harshness of punishment: *How do you consider the punishment for DUI?* The response options were Hard enough, Insufficient or Excessive. Furthermore, they were asked whether or not they changed their behavior after the punishment.

The questionnaire was used to ascribe drivers to different groups according to demographic and psychosocial characteristics, as well as to identify driving habits and risk factors.

### Demographic variables

- Gender: male or female.
- Age: 14-17, 18-24, 25-29, 30-44, 45-65 and over 65 years old.
- Educational level.
- Type of driver: professional or non-professional.
- Employment status: currently employed, retired, unemployed, unemployed looking for the first job, homemaker or student.

### Driving habits

- Frequency: the frequency with which the participant drive, the possible choices being Every day, Nearly every day, Just weekends, A few days a week, or A few days per month.
- Mileage: the total distance in number of kilometers driven or travelled weekly, monthly or annually.
- Route: type of road used regularly, including street, road, highway or motorway, and tollway.
- Car use: motives for car use, for instance, to work, to go to work and return home from work or study centre, personal, family, recreational, leisure and others.

### Experience/risk

- Experience: number of years the participant has held a driver license, grouping them as 2 years or less, 3-6, 7-10, 11-15, 16-20, 21-25, 26-30 and over 30 years.
- Traffic offenses. Number of sanctions in the past three years (none, one, two, three or more).
- Accidents. Number of accidents as driver throughout life (none, one or more than one), and their consequences (casualties or deaths, or minor damages).

Once data were collected, a number of statistical analyses were performed, using the Statistical Package for the Social Sciences (SPSS), in order to obtain relevant information according to the aims of the study.

## Results

**Figure 1:**
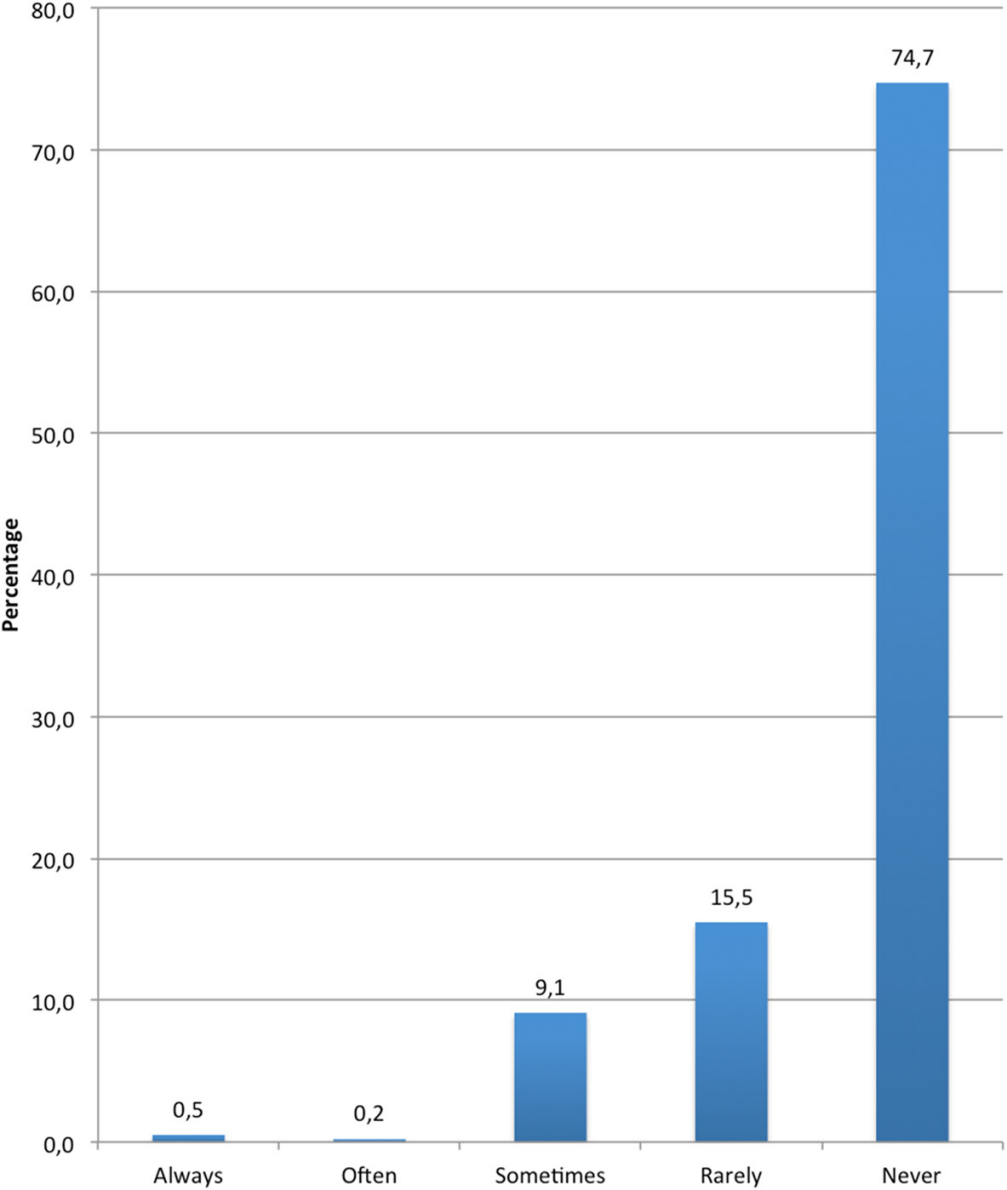
**Frequency of DUI.**

74.7% of the sample said that they had never driven under the influence. 15.5% of drivers said they did it almost never, and only the remaining 9.7% (sometimes 9,1%, often 0,2% or always 0,5%) acknowledged that they had driven after consuming alcohol (Figure 1).

**Figure 2:**
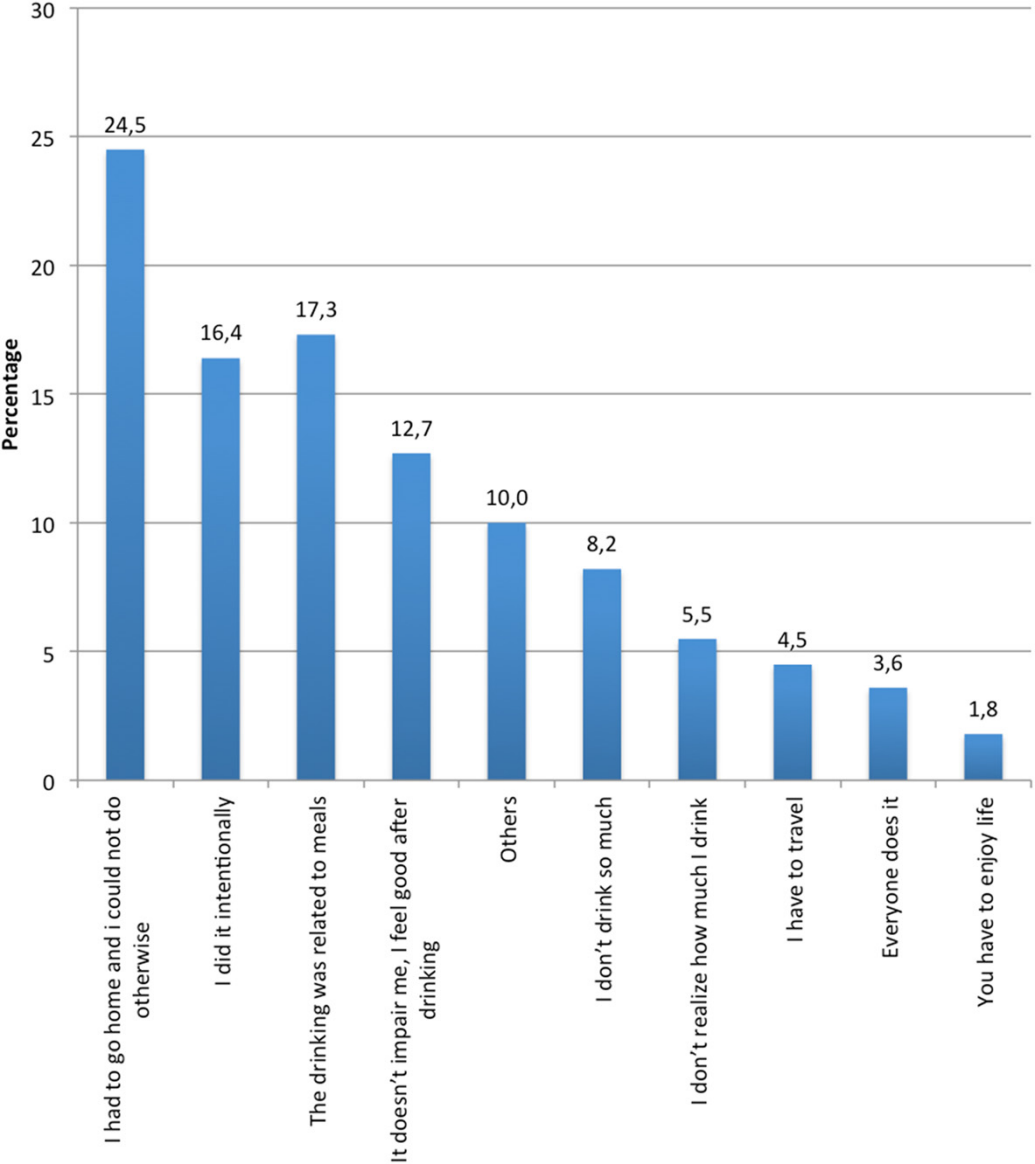
**Reasons for DUI.**

Regarding the main reasons that led the drivers to act this way, expressed among drivers who admitted to having driven under the influence of alcoholic beverages, 24.5% of them indicated that it was unavoidable, as “I had to go home and couldn’t do anything else”, while 17.3% claimed that the act of drink-driving was an unintentional consequence or “something associated with meals”, and only 16.4% admitted having done it “intentionally”. In addition, 12.7% considered that “alcohol doesn’t impair driving” anyway (Figure 2).

“In any case, 60% of the interviewees perceived driving under the influence of alcohol as the highest risk factor for traffic accidents.”

Among them, the perception of this risk (or dangerousness of driving under the influence) is greater in women [F (1, 1081) = 41.777 p <0.05], adults aged between 18 and 44 [F (5, 1075) = 4.140 p <0.05], drivers who have never been fined for this infraction [F (2, 1080) = 29.650 p <0.05], drivers who had never committed the offense [F (4, 1077) = 40.489 p <0.05], and drivers who have never been involved in an accident [F (1, 1081) = 12.296 p <0.05]. Table 1 shows the values for this perception by gender and age.

**Table 1:** **Perception of risk of DUI-related traffic accident, by drivers’ gender and age**

		**N**	**Mean**	**SD**	**gl**	**F**	**Sig**
Gender	Women	416	9.45	1.120	1.0	41.78	0.000
	Men	667	8.84	1.728			
Age	18-25	12	9.08	0.900	5.0	4.14	0.001
	26-35	111	9.22	1.516			
	36-45	136	9.21	1.388			
	46-65	361	8.94	1.683			
	>65	88	8.51	1.965			

There appears to be no significant relationship between the perceived risk attributed to DUI and other variables such as educational level, type of driver, driving frequency, vehicle use and years of experience.

The main reasons put forward for not drinking and driving included not drinking in any circumstances (50,5%), to avoid accidents (28,3%) as opposed to avoiding sanctions (10,4%) - such as financial penalties (8,4%), withdrawal of driving license (1,8%) or jail (0,2%) - or other reasons related to attitudes to road safety (16,6%).

On a scale of 0-10, participants rated the risk of economic penalties when driving under the influence of the alcohol with an average of 5.2, in other words they estimate the probability of being fined as roughly half of the times one drives drunk.

The perception of this risk (penalty or financial punishment for driving under the influence) is also greater in women [F (1, 1095) = 30,966 p <0.05], drivers who have never been involved in an accident [F (1, 1095) = 8.479 p <0.05], and drivers who had never been fined for this infraction [F (2 1094) = 12.515 p <0.05].

There appears to be no significant relationship between the perceived risk of financial penalty and other variables such as educational level, employment, type of driver, driving frequency, vehicle use and years of experience.

Almost everyone (99.1%) thinks that DUI is punishable and only 0.9% of drivers think it is not.

On a scale of 0-10, participants assigned an average of 9.1 to the need to punish this traffic breach severely. The score is higher in women [F (1, 1086) = 29.474 p <0.05], adults aged 18 to 24 years [F (5, 1089) = 2.699 p <0.05], drivers who have never been involved in an accident [F (1, 1095) = 8.479 p <0.05], and people who had never been fined for this reason [F (2, 1085) = 26,745 p <0.05], which means that these groups are less tolerant of this kind of behavior. By age, college students are the least tolerant and retirees are the most tolerant.

There was no significant relationship between the perceived need to punish this behavior harshly and variables such as type of driver, driving frequency and vehicle use.

**Figure 3:**
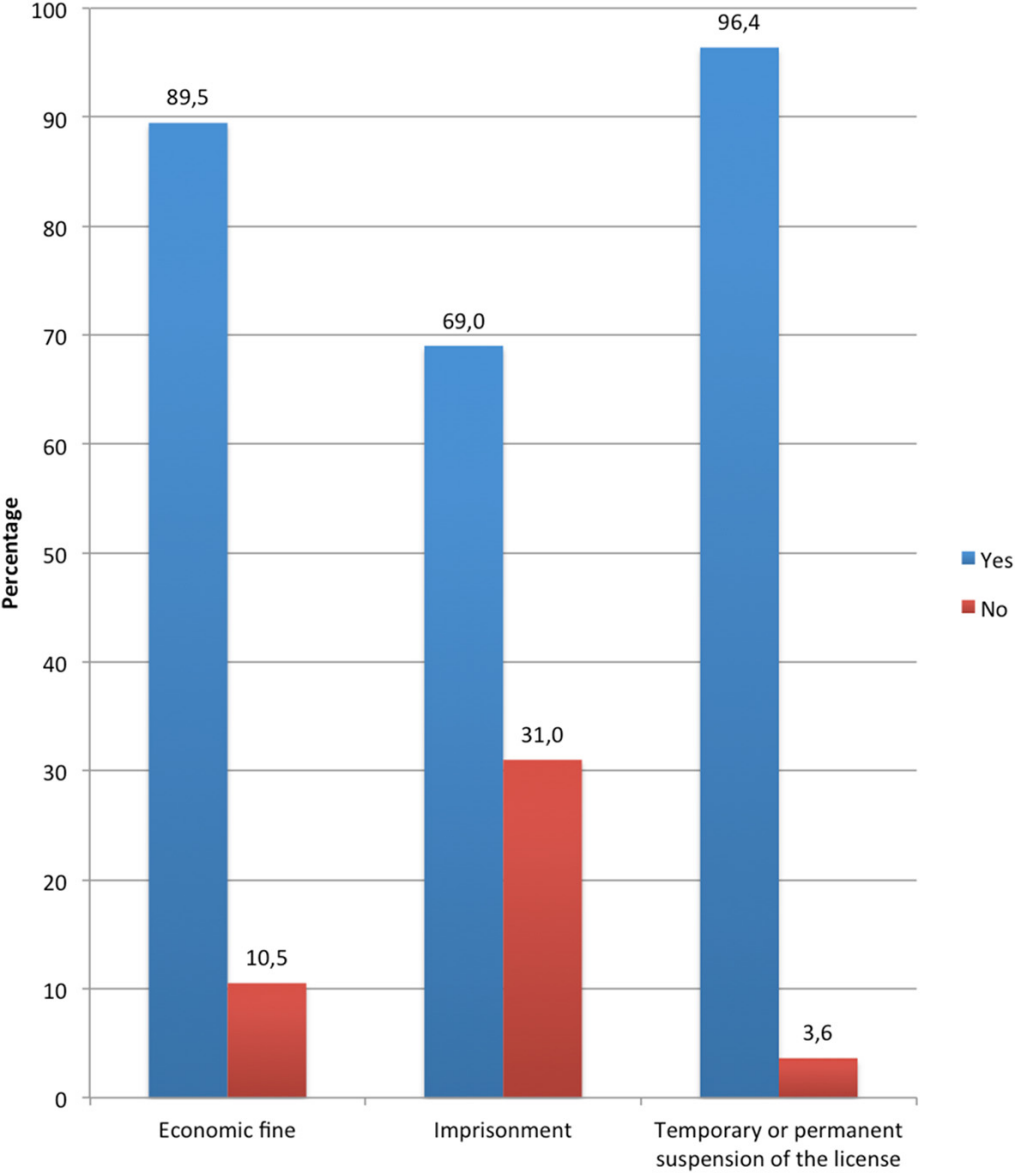
**Type of sanction the driver think DUI is subject to.**

Regarding the type of sanctions, 89.5% of drivers think that driving under the influence is subject to an economic fine, almost 70% say it could even be punished by imprisonment, while 96.4% believe it can lead to a temporary or permanent suspension of the license (Figure 3).

**Figure 4:**
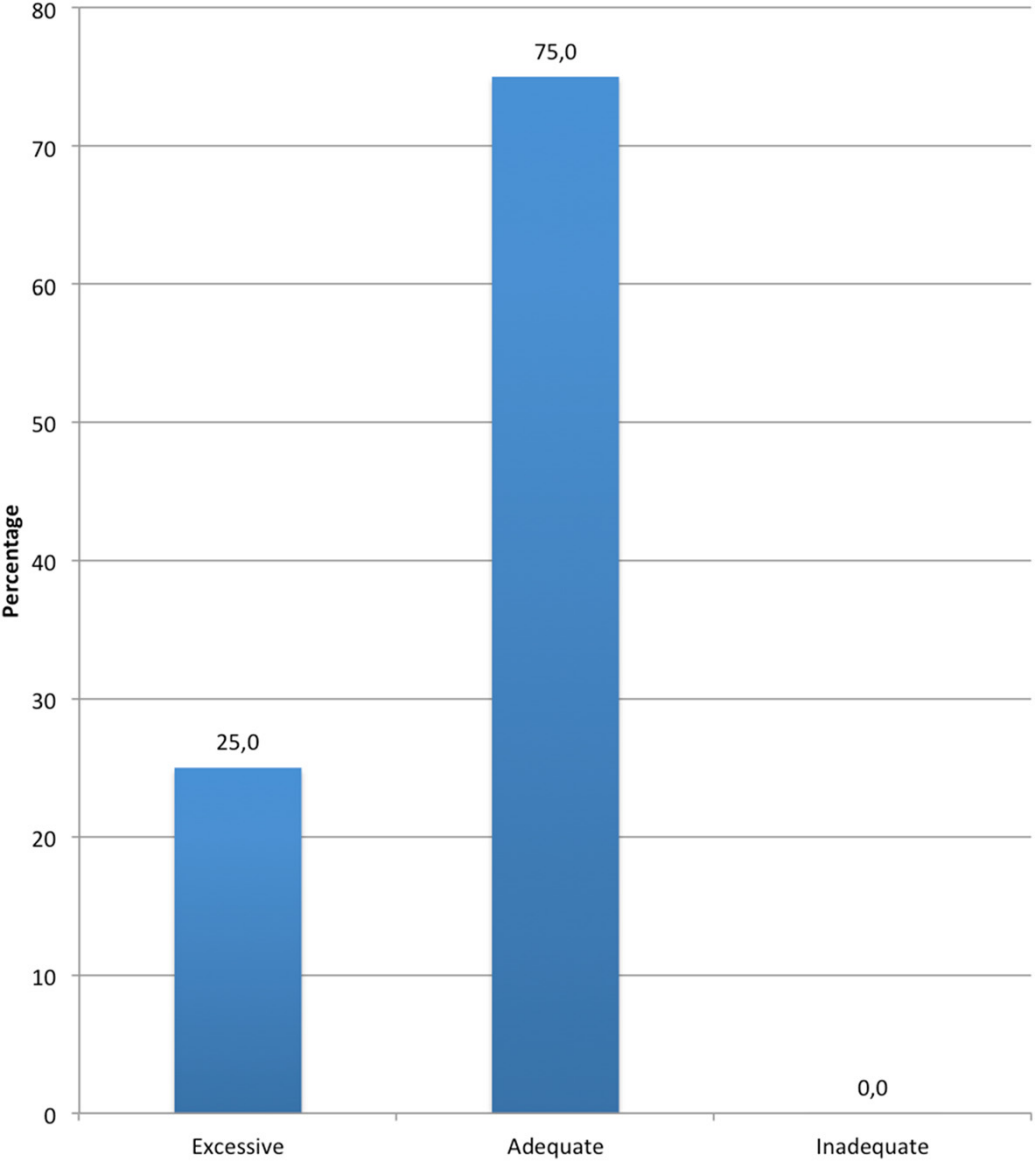
**Perception of punishment harshness imposed for DUI.**

**Figure 5:**
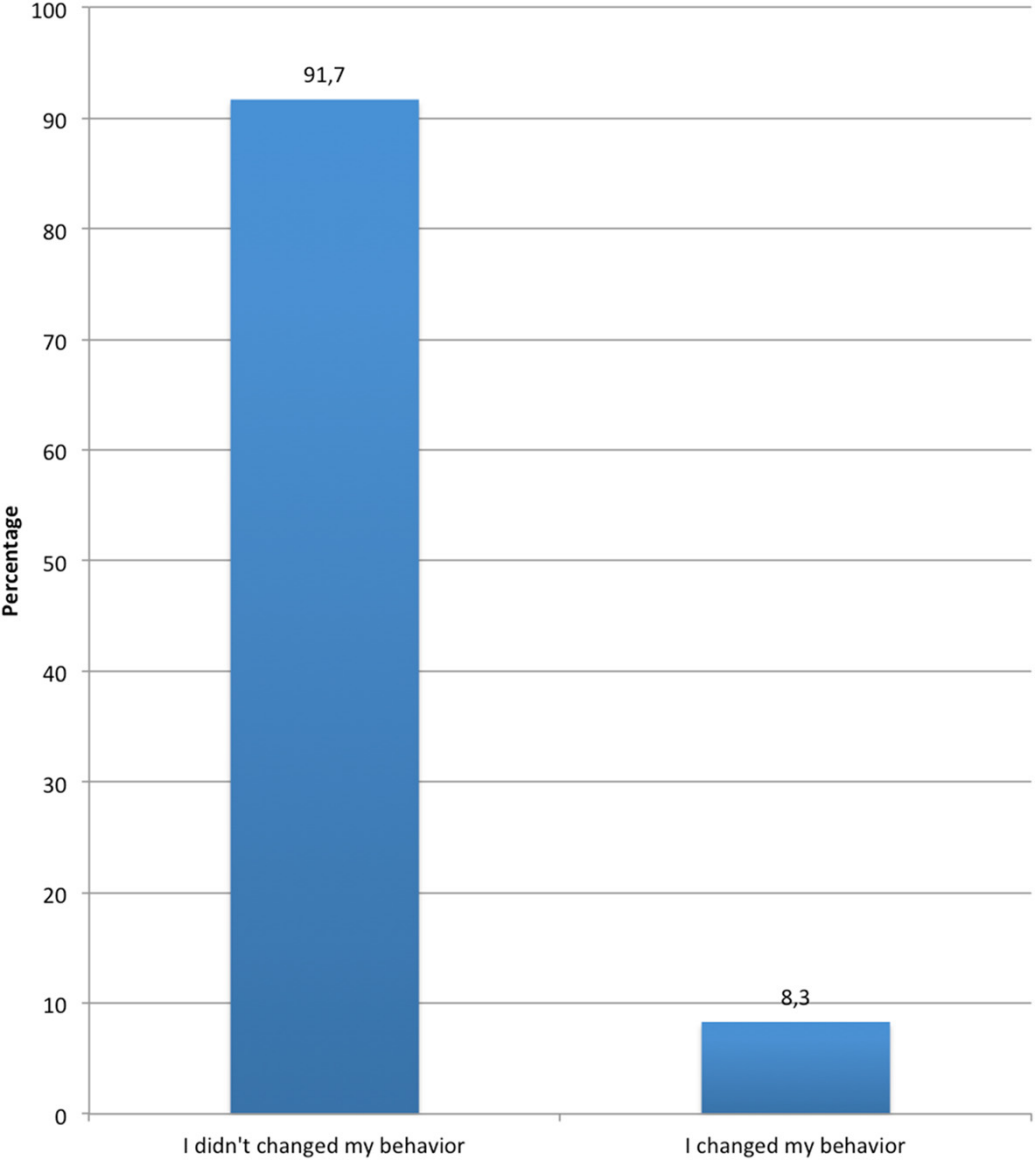
**Perception concerning behavior change after punishment for DUI.**

Among the drivers who had been fined for DUI, nearly 75% considered that the imposed punishment was adequate, while the remaining 25% saw it as excessive (Figure 4). Finally, 91.7% of this group found they had changed their behavior after punishment (Figure 5).

## Discussion

Alcohol is a major risk factor in traffic accidents. From the objective standpoint, alcohol interferes with the skills needed to drive safely, as evidenced by numerous studies on driving under the influence of alcohol conducted to date. From the subjective point of view, drivers also perceive it as dangerous, as our study shows.

Around 60% of respondents believe that driving under the influence of alcohol is maximum risk behavior. A smaller percentage compared to those reported by other studies in which the percentage of people that saw drink-driving as a major threat to safety reached 81% [15].

First, we note a clear correlation between perceived risk and avoidance behavior. In general the higher the perceived risk, the lower the probability of committing the offense, and vice versa: the lower the perceived risk, the greater the likelihood of driving after consuming alcohol.

Thus, drivers who do not commit this offense perceive that the risk of accidents associated with DUI is very high. When it comes to drivers who commit the offense occasionally, the perceived risk is lower, and when it comes to drivers who often drive under the influence of the alcohol, the perception of risk is clearly inferior. Thus, the frequency of DUI and risk perception seem to be inversely related.

These results are related to the hypothesis of optimistic bias, which states that drinkers are overly optimistic about probabilities of adverse consequences from drink. In a study [16] about overconfidence about consequences of high levels of alcohol consumption, the authors established an alternative to the optimism bias hypothesis that could explain our findings, affirming that persons who drink frequently and consume large amounts of alcohol daily could be more familiar with the risks of such behaviors.

Another important finding is that the risk perception of traffic accident as a result of DUI is influenced by variables such as sex and age. In relation to gender, the perception of risk seems to be higher in women than in men. In relation to age, risk perception is higher in adults between 18 and 44 years old.

The finding about the reason for not drinking and driving supports the already evident need for an integrative approach to developing sustainable interventions, combining a range of measures that can be implemented together. In this way, sustainable measures against alcohol and impaired driving should continue to include a mix of approaches, such as legislation, enforcement, risk reduction and education, but focus efforts more closely on strategies aimed at raising awareness and changing behavior and cultural views on alcohol and impaired driving.

Almost all the drivers surveyed are well aware that driving after drinking any alcoholic beverage is a criminal offense. They also consider that this is a type of infraction that should be punished harshly. In this respect, they assign nine points on a scale of ten possible.

Finally, with regard to the type of sanctions, 90% of drivers think that driving drunk is punishable by a fine. 96.4% consider that it may result in temporary or permanent suspension of driving license, and 70% believe that it can be punished with imprisonment.

In any case, there are several limitations of this study. This was a population-based study of Spanish drivers; there is possibly a lack of generalizability of this population to other settings.

Another possible limitation of this study is the use of self-report questionnaires to derive information rather than using structured interviews. Similarly, self-reported instruments may be less accurate than objective measures of adherence as a result of social desirability bias.

## Conclusions

In Spain, various traffic accident prevention programs have been implemented in recent years. Some of them were alcohol-focused, designed to prevent driving under the influence and to inform the Spanish population about the dangers associated with this kind of risk behavior.

As a result, many Spanish drivers seem to be sensitized to the risk of driving drunk. As revealed in our survey, many Spanish drivers never drive under the influence of alcohol, and many of them identify DUI as maximum risk behavior. This shows that a high percentage of the Spanish population know and avoid the risks of DUI.

In any case, the reality is far from ideal, and one out of four drivers has committed this offense at least once. When asked why they did it, the two major risk factors of DUI we identified were the lack of an alternative means of transport and the influence of meals on alcohol consumption. Both situations, especially the latter, occur frequently, almost daily, while it is true that the amount of alcohol consumed in the former is considerably higher and therefore more dangerous.

In addition, most drivers are aware of the dangers of driving under the influence, and they tend to avoid the risk of accident or penalty for this reason. Some drivers never drive under the influence, to avoid a possible accident. To a lesser extent, some do not drive under the influence to avoid a possible fine. They usually think that the possibility of sanction in the event of DUI is so high that they will be fined every two times they risk driving drunk.

Moreover, drivers know the legislation regulating DUI and they believe that the current penalty for DUI is strong enough. Nevertheless, even though almost all the drivers that were fined for this reason say they changed their behavior after the event, nine out of ten drivers would penalize this kind of offense even more strongly.

Knowing how alcohol consumption impairs safety and driving skills, being aware of the associated risks, knowing the traffic regulations concerning DUI and penalizing it strongly are not enough. Many drivers habitually drive after consuming alcohol and this type of traffic infraction is still far from being definitively eradicated.

Additional efforts are needed for better management of a problem with such important social and practical consequences. Efforts should be focused on measures which are complementary to legislation and enforcement, increasing their effectiveness, such as education, awareness and community mobilization; Alcolock™; accessibility to alcohol or brief interventions.

## Abbreviation

DUI: Driving under the influence
